# Effect of corticosubcortical iron deposition on dysfunction in CADASIL is mediated by white matter microstructural damage

**DOI:** 10.1016/j.nicl.2023.103485

**Published:** 2023-07-27

**Authors:** Xiuqin Jia, Yingying Li, Yunqing Ying, Xuejia Jia, Weijun Tang, Yueyan Bian, Jiajia Zhang, Danny J.J. Wang, Xin Cheng, Qi Yang

**Affiliations:** a Department of Radiology, Beijing Chaoyang Hospital, Capital Medical University, Beijing 100020, China; b Key Lab of Medical Engineering for Cardiovascular Disease, Ministry of Education, Beijing 100020, China; c Department of Neurology, National Center for Neurological Disorders, National Clinical Research Centre for Aging and Medicine, Huashan Hospital, Fudan University, Shanghai 200040, China; d Department of Radiology, Huashan Hospital, Fudan University, Shanghai 200040, China; e Laboratory of FMRI Technology (LOFT), USC Mark & Mary Stevens Neuroimaging and Informatics Institute, Keck School of Medicine, University of Southern California, Los Angeles, CA 90007, United States

**Keywords:** Quantitative susceptibility mapping (QSM), Peak width of skeletonized mean diffusivity (PSMD), CADASIL, Cerebral small vessel disease (cSVD), Iron deposition, Cognitive deficit

## Abstract

Iron dysregulation may attenuate cognitive performance in patients with CADASIL. However, the underlying pathophysiological mechanisms remain incompletely understood. Whether white matter microstructural changes mediate these processes is largely unclear. In the present study, 30 cerebral autosomal dominant arteriopathy with subcortical infarcts and leukoencephalopathy (CADASIL) patients were confirmed via genetic analysis and 30 sex- and age-matched healthy controls underwent multimodal MRI examinations and neuropsychological assessments. Quantitative susceptibility mapping and peak width of skeletonized mean diffusivity (PSMD) were analyzed. Mediation effect analysis was performed to explore the interrelationship between iron deposition, white matter microstructural changes and cognitive deficits in CADASIL. Cognitive deterioration was most affected in memory and executive function, followed by attention and working memory in CADASIL. Excessive iron in the temporal-precuneus pathway and deep gray matter specific to CADASIL were identified. Mediation analysis further revealed that PSMD mediated the relationship between iron concentration and cognitive profile in CADASIL. The present findings provide a new perspective on iron deposition in the corticosubcortical circuit and its contribution to disease-related selective cognitive decline, in which iron concentration may affect cognition by white matter microstructural changes in CADASIL.

## Highlights

• How iron deposition contributes to cognitive deficit in CADASIL remains elusive.
• Memory and executive function were predominantly identified in CADASIL.
• Iron deposition was identified in the temporo-PCU pathway and deep GM in CADASIL.
• Iron deposition was related with specific cognitive deficit in CADASIL.
• Disrupted WM microstructure measured by PSMD mediated their relationship.

## 1 Introduction

Cerebral autosomal dominant arteriopathy with subcortical infarcts and leukoencephalopathy (CADASIL), a rare hereditary cerebral small vessel disease (cSVD) caused by NOTCH3 gene mutations, is a major cause of stroke and dementia in middle-aged adults (METACOHORTS Consortium, 2016). However, the pathophysiological mechanisms underlying brain damage and cognitive dysfunction in this disease remain largely elusive. No specific targeted treatment exists that slows the progression of this disease process.

Due to an earlier age of onset, CADASIL has been recognized as an important “model” to investigate vascular cognitive impairment independent of neurodegenerative pathologies in elderly individuals. Conventional MRI in CADASIL patients shows similar features as in cSVD, including white matter hyperintensities (WMHs), cerebral microbleeds (CMBs) and enlarged perivascular spaces(ePVS), and lacunes (Chabriat et al., 2009). Pathologically, *NOTCH3* mutations in CADASIL cause degeneration or loss of vascular smooth muscle cells (VSMCs) and deposits of granular osmiophilic material (GOM) in vessel walls (Kalimo et al., 2002), leading to the accumulation of blood-derived neurotoxic substances in the brain (Montagne et al., 2015, Zhang et al., 2017).

Of note, iron in the brain is an essential element required for maintaining normal physiological functions, suggesting that the brain iron concentration must be well regulated (Singh et al., 2014). Excessive amounts of brain iron have deleterious effects on cognitive dysfunction and noncognitive manifestations in normal aging and disease states (Li et al., 2021, Piñero and Connor, 2000, Sun et al., 2020, Liem et al., 2012). To the extent that the presence of iron dysregulation may attenuate neuronal functions, the region-specific iron alteration may help explain the disease-related selective cognitive decline in CADASIL.

Brain iron accumulation has been convergently proposed as one of the pathomechanisms in CADASIL, particularly in deep gray matter (Liem et al., 2012, Sun et al., 2020). A susceptibility-weighted MRI (SWI) study has yielded reports that iron depositions in the putamen and caudate are possibly associated with clinical disease severity in CADASIL (Liem et al., 2012). As a novel MRI technique, quantitative susceptibility mapping (QSM) has allowed elucidation that iron is the dominant source of magnetic susceptibility in gray matter (Langkammer et al., 2012). Consistently, a recent study also found that increased susceptibility signals in these subcortical nuclei could be an important biomarker for CADASIL severity by using QSM (Sun et al., 2020). Furthermore, in a recent study, it was highlighted that iron deposition in the basal ganglia is implicated in cognitive dysfunction in patients with CADASIL (Uchida et al., 2020).

Of interest to our study, WMHs are commonly observed in CADASIL patients due to an interplay of hypoxia, ischemia because of altered cerebrovascular autoregulation, and blood–brain barrier leakage (Gouw et al., 2011, Abraham et al., 2016). Interestingly, iron deposition has been linked to an increased WMH burden (Gebril et al., 2011, Bergsland et al., 2017). Indicative of CADASIL, early involvement of WMHs in the anterior temporal poles has been reported to be associated with an ePVS and degeneration of myelin accompanied by a lack of drainage of the interstitial fluid (Yamamoto et al., 2009). Although the relationship of iron deposition to cognitive dysfunction has been consistently reported, the mechanisms of this relationship are largely unexplored. Given the interrelationship between iron deposition and WMH, a potential mechanism of iron dysregulation implicated in cognitive deficits in CADASIL might be involved in increased WM microstructural changes.

The results of a previous study revealed that the novel diffusion tensor imaging (DTI)-based marker of peak width of skeletonized mean diffusivity (PSMD) outperforms conventional markers and is more sensitive to disease related changes than other diffusion-based metrics in explaining cognitive impairment scores (Baykara et al., 2016). On this basis, the current study was designed to examine whole brain iron deposition in CADASIL patients using voxel-based QSM. Furthermore, we explored the potential mediating effect of the white matter microstructural changes measured by PSMD on the relationship between iron deposition and cognitive dysfunction.

## 2 Material and methods

### 2.1 Participants

In the current study, 30 CADASIL patients (13 females, mean age of 45.97 ± 14.34 years) and 30 age- and sex-matched healthy volunteers (8 females, mean age of 43.33 ± 12.12 years) were recruited. The inclusion criteria for CADASIL patients were confirmed genetic diagnosis of typical mutation of the NOTCH3 gene. Participants with any history of head injury, alcoholism, drug abuse, or severe psychiatric illness that might impair cognition were excluded. This study was approved by the Institutional Review Board and Ethics Committee at Beijing Chaoyang Hospital of Capital Medical University and Huashan Hospital of Fudan University. Written informed consent was obtained from each participant.

### 2.2 Clinical and neuropsychological assessments

The demographic and clinical characteristics of age, sex, vascular risk factors, transient ischemic attack (TIA)/stroke, and headache were recorded. All patients underwent a modified Rankin Scale (mRS) assessment to evaluate their degree of disability/dependence. Comprehensive neuropsychological assessments were conducted to calculate a composite cognitive score of performance in the cognitive domains including the following: (i) Rey’s auditory verbal learning test (AVLT) (immediate and delayed recall) for memory; (ii) Stroop interference response time and trail making test (TMT) A and B within the executive domain; (iii) Symbol digit modalities test (SDMT) and digit span test (forward and backward) within the attention and working memory domain; (iv) Rey’s figure copy and memory within visuospatial abilities; and (v) Boston naming and verbal fluency within language function. Scores obtained from assessing these cognitive domains were converted to *z* scores and summed to generate a composite indicator of memory, executive functioning, attention and working memory, visuospatial ability, and language performance *(*Andrade, 2021*)*.

### 2.3 MRI data acquisition

MRI data were acquired using a Siemens 3-Tesla Prisma MRI system (Siemens, Erlangen, Germany) using a 64-channel head coil. Foam padding and headphones were used to limit head motion and reduce scanner noise. Participants were instructed to keep still and remain motionless. We acquired a 3D T1-weighted structural magnetization-prepared rapid gradient echo (MPRAGE) with parameters of TR = 2,300 ms, TE = 2.98 ms, flip angle = 9°, and 1 mm^3^ isotropic voxels. T2-weighted fluid-attenuated inversion recovery images were acquired using parameters of TR = 5,000 ms, TE = 388 ms, flip angle = 120°, and 1 mm^3^ isotropic voxels. We acquired diffusion-weighted images with parameters of TR = 8,600 ms, TE = 68 ms, flip angle = 120°, 2 mm × 2 mm in-plane resolution with 80 slices of 2 mm, diffusion-weighting along 45 gradient directions with a b0-value of 1000 s/mm^2^. MR magnitude and phase data were acquired with a gradient-echo sequence (GRE) with the following parameters: TR = 28 ms, TE = 20 ms, flip angle = 15°, 0.9 mm × 0.9 mm in-plane resolution with 88 slices of 0.9 mm.

### 2.4 Data preprocessing

Field inhomogeneities of MRI data were corrected via the ANT-based toolkit N4BiasField Correction to correct nonuniform image intensities of the same tissue class.

#### 2.4.1 cSVD imaging characteristics analysis

The typical imaging characteristics of CSVD (WMHs, CMBs, ePVS, and lacunes) were defined as previously described (Wardlaw et al., 2013). WMHs were segmented by the lesion prediction algorithm as implemented in the LST toolbox (https://www.statistical-modeling.de/lst.html) based on FLAIR images using T1 as a reference. Normalized WMH volume was calculated to account for the whole brain volume. Normal-appearing white matter (NAWM) was generated by extracting the WMH from the WM. CMBs were recorded as binary variables, indicating the presence (1) or absence (0) (Hong et al., 2023). The total cSVD score was used to evaluate the disease burden of CADASIL, ranging from 0 to 4, by counting four features of cSVD on MRI, including WMH, lacunes, microbleeds, and enlarged perivascular space (Staals et al., 2014)*.*

#### 2.4.2 QSM analysis

QSM processing was calculated from the phase and magnitude images using KKI QSM toolbox v3.0 (JHU/KKI *QSM* Toolbox V3.0) (Bao et al., 2021, Bao et al., 2016) and SPM12 (https://www.fil.ion.ucl.ac.uk/spm). A brain mask was obtained by T1-weighted imaging using FSL’s brain extraction tool (BET, FMRIB Oxford, UK). Phase unwrapping was performed using the Path method (Abdul-Rahman et al., 2005). Subsequently, the projection onto the dipole field (PDF) method (Liu et al., 2011a) was used to eliminate background fields. Then, inverse dipole calculations to obtain the susceptibility maps were performed using MEDI (Liu et al., 2011b, Liu et al., 2012).

Next, the calculated QSM images in the individual spaces were coregistered to T1 images through the magnitude image. Subsequently, T1 images were segmented and normalized into MNI space based on tissue probability maps (TPMs). Finally, the spatially normalized QSM maps were smoothed with an 8 mm FWHM isotropic Gaussian kernel. Based on the fact that brain iron in gray matter is a key contributor to the susceptibility of QSM (Hametner et al., 2018), to minimize the confounding effect of myelin density on QSM measurement (Langkammer et al., 2012), the cortical and deep gray matter regions were considered in the present study.

#### 2.4.3 PSMD processing

**Fig. 1:**
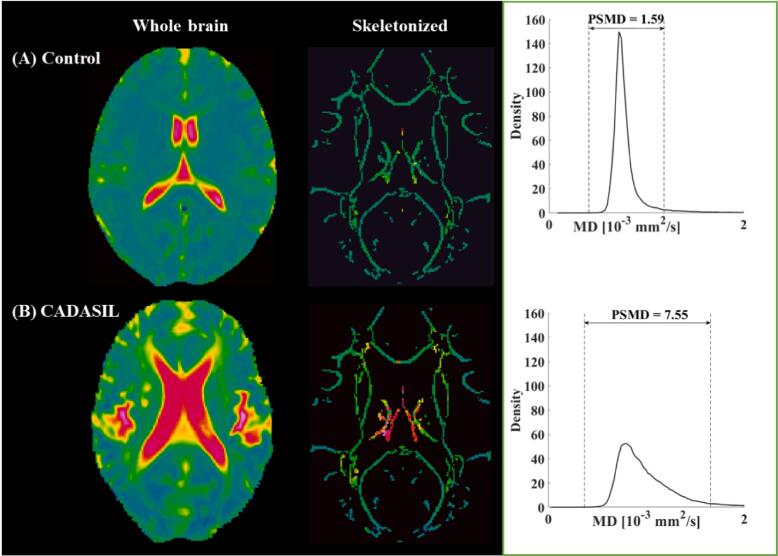
Skeletonization and histogram analysis. Examples of MD maps from one healthy control (A) and one CADASIL patient (B) projected onto the standard skeleton. The peak width of skeletonized mean diffusivity (PSMD) is calculated as the difference between the 95th and 5th percentiles.

Diffusion-weighted images were corrected for eddy currents and head motion using the FMRIB Software Library (FSL; http://www.fmrib.ox.ac.uk/fsl/). Diffusion tensors and scalar diffusion parameters of FA and MD were calculated using DTIFIT (FSL). DTI data were skeletonized using the tract-based spatial statistics procedure. As described in a previous study (Baykara et al., 2016), FA data were normalized into the standard space FMRIB-1 mm-FA template and projected onto the skeleton. By using the FA-derived projection parameters, MD images were then projected onto the skeleton. Finally, to avoid a partial volume effect, the final MD skeletons were further masked with the template skeleton threshold at an FA value of 0.3. PSMD was calculated as the difference between the 95th and 5th percentiles of the voxel-based MD values within the skeleton (Fig. 1).

#### 2.4.4 Voxel-based morphometry (VBM) analysis

To rule out the effect of gray matter volume on iron levels, a VBM analysis was performed to measure gray matter volume in the CADASIL group and healthy controls.

VBM was conducted using the Computational Anatomy Toolbox (CAT12, http://dbm.neuro.uni-jena.de/cat) to compare brain structural alterations between healthy controls and patients with CADASIL. T1-weighted images were segmented using the unified segmentation model into gray matter (GM), white matter (WM), and cerebrospinal fluid (CSF) based on tissue probability maps in Montreal Neurological Institute (MNI) space. The spatially normalized GM maps were modulated by the Jacobian determinant of the deformation field and corrected for individual brain sizes. The modulated and normalized GM images (voxel size: 1.5 × 1.5 × 1.5 mm^3^) were smoothed with an 8 mm full width at half maximum (FWHM) isotropic Gaussian kernel.

### 2.5 Statistical analysis

Normality of clinical and neuropsychological data was assessed by the Kolmogorov–Smirnov (KS) test to choose appropriate parametric and nonparametric tests using SPSS v22. Independent two-sample *t* tests (for the parametric test) or Mann–Whitney *U* tests (for the nonparametric test) were performed to compare changes between the two groups. Significance was determined by *p* < 0.05.

The cognitive impairment in CADASIL was defined based on the detailed cognitive battery: expected z scores for each test and each subject were calculated based on a multiple regression analysis performed in the healthy group adjusted for age, sex, and education (*Aarsland et al., 2009*). CADASIL patients were defined as cognitive impairment when the actual z score for a given test was at least 1.5 SD lower than the expected score in at least two tests in one domain or at least one test in at least two domains (Caviness et al., 2007)*.*

Regional differences in VBM (based on the whole brain) and QSM (based on GM) between the two groups were assessed using the general linear model on a voxel wise level based on SPM12. The results were reported based on an uncorrected voxelwise height threshold of *p* < 0.001 combined with an FWE-corrected clusterwise threshold of *p* < 0.05.

Correlation of iron deposition and the presence of CMBs, cSVD burden, and clinical disability was analyzed using Spearman rank correlation, the Bonferroni correction was applied to correct for the number of brain areas with abnormal iron accumulation (*p* = 0.05/ number of brain areas with abnormal iron accumulation).

For iron deposition, PSMD, and cognitive performance, we first analyzed the relationship between iron level in brain areas with abnormal iron accumulation, PSMD, and the cognitive subdomains, also using the Bonferroni correction to correct for the number of brain areas with abnormal iron accumulation. If iron deposition in specific brain regions, PSMD, and specific cognitive sub-domains are correlated with each other, the mediation analysis will be performed.

Mediation analyses were tested using PROCESS for SPSS v22 (Hayes, 2012). The regression analysis between the independent variable (i.e., iron deposition) and dependent variable (i.e., cognitive functioning) was first performed. Next, the direct effects of the predictor (i.e., iron deposition) on the mediator (i.e., PSMD) and the direct relationship between the mediator and cognitive functioning were tested. Finally, we tested the indirect mediating effect, or the extent to which the relationship between iron deposition and cognitive functioning operated statistically through PSMD.

## 3 Results

### 3.1 Clinical and neuropsychological characteristics

Demographic data, vascular risk factors, clinical and imaging characteristics of the healthy controls and patients with CADASIL are shown in Table 1. There were no significant differences between patients with CADASIL and healthy controls in age (*p* = 0.46), sex (*p* = 0.28), or education (*p* = 0.16). The mutation spectrum at the protein level is listed in Supplementary Table S1.

**Table 1:** Comparison of demographic and clinical characteristics between healthy controls and patients with CADASIL.

	**CADASIL** **(*n* = 30)**	**Controls** **(*n* = 30)**	***P* value**
**Demographic characteristics**			
Age, yr, mean (SD)	45.97 (14.34)	43.33 (12.12)	0.46
Female, no. {%}	13 {43.33}	8 {26.67}	0.28
Education, mean (SD)	11.07 (4.75)	12.92 (5.20)	0.16
**Vascular risk factors, no. {%}**			
Smoker	9 {30.00}	3 {10.00}	0.05
Hypertension	1 {3.33}	1 {3.33}	1.00
Hypercholesterolemia	1 {3.33}	5 {16.67}	0.92
Diabetes	1 {3.33}	0 {0.00}	0.31
**Symptoms, no. {%}**			
TIA/stroke	8 {26.67}	0 {0.00}	0.002
Headache	9 {30.00}	0 {0.00}	0.001
**Clinical test score**			
mRS, median (IQR)	0.00 (1.00)	/	/
BI index, median (IQR)	100.00 (2.40)	100.00 (0.00)	0.015
IADL, median (IQR)	23.00 (3.50)	23.00 (0.00)	0.001
**Imaging characteristics**			
Normalized WMHV, %, mean (SD)	1.82 (1.15)	0.02 (0.05)	< 0.001
Lacunar infarcts, no. {%}	16 {53.33}	0 {0.00}	< 0.001
Microbleeds, no. {%}	18 {60.00}	0 {0.00}	< 0.001
ePVS, no. {%}	15 {50.00}	0 {0.00}	< 0.001
SVD score, median (IQR)	2 (2)	/	/
PSMD, 10^-4^ mm^2^/s, mean (SD)	4.77 (2.07)	1.91 (0.18)	< 0.001

***Note***: Data are presented as the mean (SD) or median (IQR). Abbreviations: BI Index = Barthel index; IADL = Instrumental Activities of Daily Living Scale; IQR, interquartile range; mRS = modified Rankin Scale; NAWMV, normal appearing white matter volume; NIHSS = National Institute of Health Stroke Scale; PSMD = peak width of skeletonized mean diffusivity; SVD = small vessel disease; TIA = transient ischemic attack; WMHV = white matter hyperintensity volume; SD = standard deviation.

The most affected cognitive domains in CADASI were memory (Cohen’s *d* = 1.01) and executive function (Cohen’s *d* = 1.00), followed by attention and working memory (Cohen’s *d* = 0.90) (Table 2). Visuospatial ability and language were partially impaired.

**Table 2:** Comprehensive neuropsychological assessments.

**Neuropsychological tests**	**CADASIL** **(*n* = 30)**	**Controls** **(*n* = 30)**	***P* value**
**Memory: Cohen's *d* = 1.01**			
AVLT-immediate recall, mean (SD)	4.87 (2.41)	6.88 (1.82)	0.001
AVLT-delayed recall, mean (SD)	4.79 (3.26)	7.81 (2.22)	< 0.001
**Executive function: Cohen's *d* = 1.00**			
Stroop time, sec, mean (SD)	61.86 (28.57)	42.50 (11.57)	0.002
TMT (B-A), sec, mean (SD)	81.15 (38.29)	58.37 (25.56)	0.02
**Attention and working memory: Cohen's *d* = 0.90**			
SDMT, mean (SD)	37.32 (21.08)	47.85 (14.78)	0.06
Digit span total, mean (SD)	8.69 (2.97)	12.73 (3.77)	< 0.001
**Visuospatial ability: Cohen's *d* = 0.88**			
Rey complex figure-copy, median (IQR)	31.42 (4.10)	34.62 (1.76)	0.003
Rey complex figure-recall, mean (SD)	16.27 (9.81)	19.00 (7.60)	0.30
**Language: Cohen's *d* = 0.63**			
Boston, mean (SD)	22.92 (6.76)	23.72 (4.07)	0.59
Verbal fluency, mean (SD)	35.20 (16.35)	52.44 (12.62)	< 0.001

***Note***: Data are presented as the mean (SD) or median (IQR). Abbreviations: AVLT, Rey’s auditory verbal learning test; IQR, interquartile range; SD = standard deviation; SDMT, symbol digit modalities test; TMT, trail making test.

### 3.2 VBM and PSMD results

Analysis of VBM revealed that no voxel survived a corrected threshold between groups. As listed in Table 1, significantly increased PSMD (*p* < 0.001) was detected in patients with CADASIL (4.77 ± 2.07) compared to healthy controls (1.91 ± 0.18).

### 3.3 Voxel-based QSM results

**Fig. 2:**
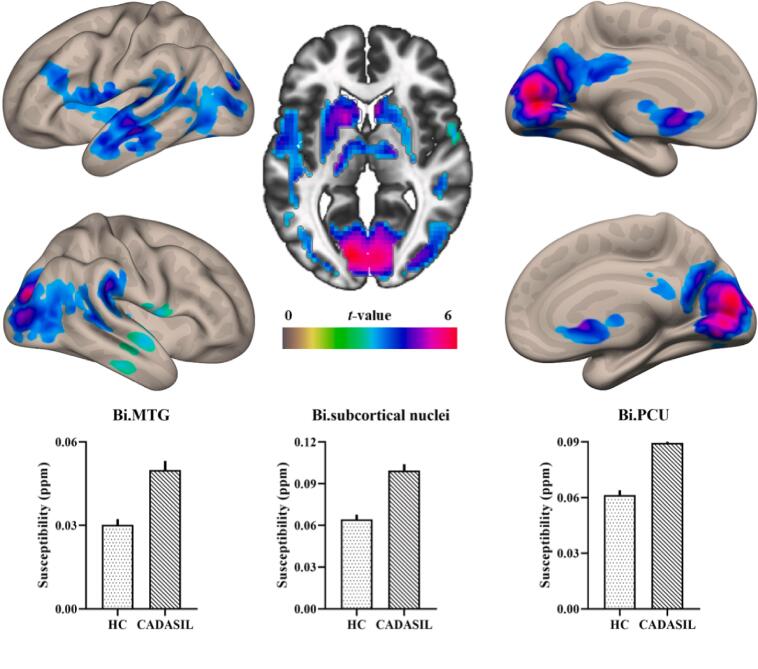
Surface and axial views of corticosubcortical iron deposition in CADASIL patients compared to healthy controls. The bar graph shows the mean extracted susceptibility values between groups in these regions.

Voxelwise comparison of the gray matter results showed that substantial iron deposition was revealed in the bilateral deep gray matter, including the caudate, putamen and thalamus, bilateral middle temporal gyrus (MTG), and bilateral precuneus extending into the lateral occipital gyrus (PCU/LOC) (Table 3 and Fig. 2). These regions were defined as regions of interest (ROIs), and the mean susceptibility within each of these ROIs was extracted. The susceptibility values of these ROIs were used for correlation analysis with PSMD, cognition, and further mediation analysis.

**Table 3:** Increased susceptibility in CADASIL compared to healthy controls.

**Anatomical regions**	**Cluster size (voxel)**	**MNI**			**Peak *T* value**
		**x**	**y**	**z**	
Bi.Caudate/Putamen/Thalamus	914	9	18	12	5.31
Bi.PCU/LOC	3042	12	−87	12	6.39
Lt.MTG	1097	−57	−18	−12	4.86
Rt.MTG	133	54	−18	−21	3.25

***Note***: The results were thresholded based on an uncorrected voxelwise height threshold of *p* < 0.001 combined with an FWE-corrected clusterwise threshold of *p* < 0.05. Abbreviations: LOC, lateral occipital cortex; PCU, precuneus; MTG, middle temporal cortex; Bi, bilateral; Lt, left; Rt, right.

### 3.4 Associations among iron deposition and the presence of CMBs, cSVD burden, and clinical disability

Correlation analysis showed that iron deposition in the bilateral deep gray matter (*r* = 0.52, *p* = 0.003) and PCU/LOC (*r* = 0.52, *p* = 0.003) was significantly associated with the presence of CMBs; iron deposition in the bilateral deep gray matter (*r* = 0.51, *p* = 0.004), PCU/LOC (*r* = 0.50, *p* = 0.005) and left MTG (*r* = 0.49, *p* = 0.006) was significantly associated with higher CSVD score; iron deposition in the and left MTG (*r* = 0.46, *p* = 0.011) was significantly associated with higher mRS score, the details are shown in Supplementary Table S2.

### 3.5 Associations among iron deposition, PSMD, and cognition

Correlation analyses showed that PSMD was significantly associated with memory (*r* = -0.70, *p* < 0.001), executive function (*r* = 0.74, *p* < 0.001), attention and working memory (*r* = -0.63, *p* = 0.002), and language (*r* = -0.65, *p* < 0.001). Significant correlations were identified between iron deposition in the bilateral deep gray matter and executive function (*r* = 0.52, *p* = 0.016) and PSMD (*r* = 0.58, *p* = 0.001), iron deposition in the bilateral MTG and executive function (*r* = 0.72, *p* < 0.001), attention and working memory (*r* = -0.50, *p* = 0.01), and PSMD (*r* = 0.55, *p* = 0.007), and iron deposition in the PCU extending into the lateral occipital cortex and attention and working memory (*r* = -0.54, *p* < 0.005) and PSMD (*r* = 0.58, *p* = 0.001).

### 3.6 Mediation analyses results

**Fig. 3:**
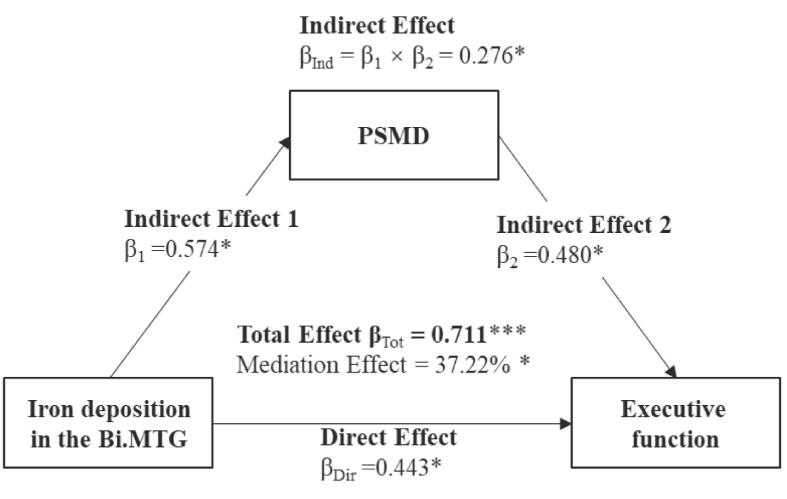
Mediation effect of peak width of skeletonized mean diffusivity (PSMD) on the relationship between iron deposition in the bilateral middle temporal gyrus (Bi.MTG) and executive function.

The direct and mediated effects of iron deposition and PSMD are presented in Table 4. Higher iron deposition was associated with lower cognitive performance and higher PSMD. The mediating effect of PSMD was significant for the relationship between iron deposition in the deep gray matter and executive function, between iron deposition in the bilateral MTG and executive function, and attention and working memory (Table 4). Of note, only the relationship between iron deposition in the MTG and executive function was partially mediated by PSMD, which means that iron deposition in the MTG has a direct effect on executive function (Fig. 3).

**Table 4:** Direct and indirect effects of iron deposition (i.e., predictor) and PSMD (i.e., mediator) on cognitive functioning (i.e., dependent variable). Separate models were run for each cognitive domain as dependent variables.

**Model effect (standard β)**		**Executive function**	**Attention & Working memory**
**Predictor: QSM in the Bi.deep gray matter**			
**Direct effect**	QSM ─> Cognition	0.095	/

	QSM ─> PSMD	0.633**	
PSMD ─> Cognition	0.674**		
**Indirect effect**	QSM ─> PSMD ─> Cognition	0.427**
**Predictor: QSM in the Bi.MTG**			
**Direct effect**	QSM ─> Cognition	0.444*	−0.269
	QSM ─> PSMD	0.574*	0.455*
	PSMD ─> Cognition	0.480*	−0.503*
**Indirect effect**	QSM ─> PSMD ─> Cognition	0.276*	−0.229*
**Predictor: QSM in the Bi.PCU**			
**Direct effect**	QSM ─> Cognition	/	−0.204
	QSM ─> PSMD		0.683***
	PSMD ─> Cognition		−0.487*
**Indirect effect**	QSM ─> PSMD ─> Cognition		−0.332

***Note***. *, *p* < 0.05; **, *p* < 0.005; ***, *p* < 0.001.

To perform mediated analysis needs to meet this condition: iron deposition in specific brain regions, PSMD, and specific cognitive sub-domains are correlated with each other; “/” represents that the combination fails to satisfy the condition to perform a mediation analysis.

Abbreviations: Bi, bilateral; MTG, middle temporal gyrus; PCU, precuneus; PSMD, peak width of skeletonized mean diffusivity; QSM, quantitative susceptibility mapping.

## 4 Discussion

The current study was designed to study brain iron deposition in the voxel wise level and its association with cSVD score, clinical disability, and cognitive performance in patients with CADASIL. Furthermore, we sought to investigate whether white matter microstructural changes mediated the relationship between iron deposition and cognitive performance. Consistent with previous findings, the current study presented a similar profile of cognitive deficits in executive function, memory, and attention and working memory in CADASIL patients (Buffon et al., 2006). Brain iron deposition in the corticosubcortical regions was detected in CADASIL patients and was a significant predictor of the cognitive manifestations of CADASIL. In support of dysfunction in cortical-subcortical circuits (Alexander et al., 1986), we found that white matter microstructural changes measured by PSMD partially/fully mediated the relationship between iron deposition in these altered regions and specific cognitive deficits. These results highlight the essential role of iron deposition and white matter microstructural changes in cognitive deterioration in CADASIL, suggesting potential insight into iron-related therapeutic strategies in this disease.

Iron deposition in the deep gray matter is frequently observed in the elderly population (Milton et al., 1991, Bilgic et al., 2012) and neurodegenerative processes (Thomas et al., 2020) and is also consistently reported in patients with CADASIL (Liem et al., 2012, Sun et al., 2020, Uchida et al., 2020). The basal ganglia-thalamic circuit has long been primarily implicated in motor behavior (Rodriguez-Oroz et al. 2009). There is extensive evidence indicating that these circuits also play an important role in cognition, particularly in executive function (*Niemann, et al., 2014*), and attention and working memory (*Moore et al., 2013*). The current study found that patients with CADASIL exhibited excessive iron accumulation in the deep gray matter compared to healthy controls. Indeed, iron in the brain is heterogeneously distributed and mainly concentrated in the basal ganglia (Haacke et al., 2005), making their function potentially susceptible to changes in iron status. This may explain the observation that iron content is linked to cognitive deficits in executive function.

To date, brain iron deposition in cortical regions is still undetermined. Early involvement of white matter hyperintensities in the anterior temporal poles has been identified as highly suggestive of CADASIL (*O’Sulivan et al., 2001;* Yamamoto et al., 2009). Extensively, in the current study, CADASIL patients exhibited significant iron deposition in the temporal cortex, which significantly contributed to deficits in executive function and attention and working memory. Interestingly, susceptibility values in the PCU extending into the lateral occipital cortex showed significantly higher iron levels in CADASIL patients than in healthy controls. Emerging evidence suggests that the PCU is critical for episodic memory retrieval, especially with autobiographical content and visuospatial imagery (Cavanna & Trimble, 2006). Dysfunction in the PCU has been consistently reported in MCI patients with cSVD (*Papma et al., 2013*) and even in elderly patients with a high vascular burden (Mayda et al., 2011). In a previous study, it was demonstrated that the presence of cSVD influences white matter integrity (*Rocca et al., 2010*), resulting in cognitive failure (Anticevic et al., 2010). The longitudinal fasciculus from the PCU connects to the lateral temporal cortex (Tanglay et al., 2022), which supports working memory encoding and maintenance (Faraco et al., 2013). Within this context, the current finding may support the idea that iron deposition in the temporo-PCU pathway in CADASIL patients is responsible for attention and working memory performance.

Previous studies suggest that iron deposition in the deep gray matter is positively correlated with the number of CMBs in CADASIL patients (Sun et al., 2020). CMBs can lead to the production of paramagnetic hemosiderin (Martinez-Ramirez et al., 2014). Glymphatic clearance dysfunction leads to the lack of prompt clearance of these hemosiderin micropools from the tissue, which may cause abnormal iron deposition in the brain (Zhou et al., 2020). Consistently, the present study shows that iron deposition in the deep gray matter and PCU/LOC is correlated with the presence of CMBs. In addition, correlation analysis also shows that iron deposition in the bilateral MTG was not correlated with the presence of CMBs. This finding suggests that in addition to untimely clearance of paramagnetic hemosiderin, there are several possible causes of iron deposition in CADASIL patients, such as blood–brain barrier (BBB) destruction (Kalimo et al., 2002, Dziewulska and Lewandowska, 2012, Li et al., 2023) and NOTCH3 mutation (Montagne et al., 2015, Zhang et al., 2017), the pathophysiological mechanism contributing to iron deposition in CADASIL remains to be elucidated.

Consistent with previous studies, the present results demonstrate that iron deposition in the deep gray matter is positively associated with cSVD burden. This finding supports the idea that excess iron deposition in deep gray matter may be a potential biomarker for CADASIL severity (Sun et al., 2020). In addition to deep gray matter, the current results also suggest that iron deposition in the cortical gray matter is also positively associated with disease burden and clinical disability in CADASIL patients. The present study indicates that iron deposition in the gray matter, whether in the deep or cortical gray matter, has the potential as a predictor of disease burden and clinical disability in patients with CADASIL.

It has been suggested that iron deposition in the caudate and putamen contribute to white matter microstructural changes in CADASIL patients (Hong et al., 2022). The current findings extend this idea by revealing that the relationship between iron deposition in the corticosubcortical region and specific cognitive deficits is fully/partially mediated by PSMD. Pathologically, excessive brain iron levels damage brain tissues by disrupting protein synthesis or increasing vulnerability to oxidative stress (Gaasch et al., 2007). Accordingly, we found that iron deposition in the corticosubcortical region worsened clinical symptoms independently of morphometric deformation.

We identify several study limitations that should be acknowledged. To minimize the confounding effect of myelin density on QSM measurement, only the gray matter was considered in the present study. Thus, caution should be taken when interpreting magnetic susceptibility differences as iron-related changes as other metals may also cause subtle susceptibility changes. In addition, BBB integrity and cerebral perfusion could influence the changes in QSM values, and the combination of arterial spin labeling and dynamic contrast-enhanced-MRI in future studies could help to clarify the relationship between iron deposition and BBB integrity and cerebral blood flow. Finally, longitudinal studies would help to clarify the causal relationship between region-specific altered iron levels and cognitive manifestations mediated by disruption of white matter microstructural integrity in CADASIL and determine to what extent the current findings may be generalized.

In conclusion, we identified region-specific iron concentration alterations in CADASIL patients related to cognitive status independent of morphometrical deformation. Going beyond existing knowledge, the current findings shed new light on the mechanisms triggered by iron alterations that influence cognitive manifestation in CADASIL. They extend our exploration of the mediation effect of damage to white matter microstructural integrity and provide a novel perspective on improved understanding of disease etiology.

## CRediT authorship contribution statement

**Xiuqin Jia:** Data curation, Software, Validation, Writing – original draft. **Yingying Li:** Data curation, Software, Validation, Writing – original draft. **Yunqing Ying:** Data curation. **Xuejia Jia:** Data curation. **Weijun Tang:** . **Yueyan Bian:** Methodology. **Jiajia Zhang:** Methodology. **Danny J.J. Wang:** Methodology, Writing – review & editing. **Xin Cheng:** Conceptualization, Formal analysis, Resources, Project administration. **Qi Yang:** Methodology, Project administration, Supervision, Writing – review & editing.

## Declaration of Competing Interest

The authors declare that they have no known competing financial interests or personal relationships that could have appeared to influence the work reported in this paper.

## Data Availability

Data will be made available on request.
