# Supplementary material for: Effect of corticosubcortical iron deposition on dysfunction in CADASIL is mediated by white matter microstructural damage
Source: Neuroimage Clin. 2023 Jul 27;39:103485. doi: 10.1016/j.nicl.2023.103485 (PMC10407949; doi:10.1016/j.nicl.2023.103485)
Supplement: Supplementary data 1 [file mmc1.docx]

**Supplementary Table S1.** NOTCH3 mutations in CADASIL patients.

| **DNA** | **Protein** | **Frequency** |
| --- | --- | --- |
| c.1819C>T | p.Arg607Cys | 9 |
| c.1630C>T | p.Arg544Cys | 3 |
| c.499C>T | p.Pro167Ser | 3 |
| c.421C>T | p.Arg141Cys | 3 |
| c.268C>T | p.Arg90Cys | 2 |
| c.328C>T | p.Arg110Cys | 1 |
| c.505C>T | p.Arg169Cys | 1 |
| c.544C>T | p.Arg182Cys | 1 |
| c.3226C>T | p.Arg337Cys | 1 |
| c.2038C>T | p.Arg680Cys | 1 |
| c.401G>A | p.Cys134Tyr | 1 |
| c.1010A>G | p.Tyr337Cys | 1 |
| c.709G>A | p.Val237Met | 1 |
| c.1645C>T | p.Cys549Ser | 1 |
| c.226T>C | p.Cys76Arg | 1 |

Supplementary Table S2 Associations among iron deposition and the presence of CMBs, cSVD burden (CSVD score), and clinical disability (mRS score) in CADASIL patients.

|  |  | Bi.Caudate/Putamen/  Thalamus | Bi.PCU/LOC | Lt.MTG | Rt.MTG |  |
| --- | --- | --- | --- | --- | --- | --- |
|  |  |  |  |  |  |  |
| the presence of CMBs | *p* value | 0.003 | 0.007 | 0.068 | 0.372 |  |
|  | *r* value | 0.52** | 0.48** | 0.34 | 0.17 |  |
| CSVD score | *p* value | 0.004 | 0.005 | 0.006 | 0.133 |  |
|  | *r* value | 0.51** | 0.50** | 0.49** | 0.28 |  |
| mRS score | *p* value | 0.077 | 0.114 | 0.011 | 0.039 |  |
|  | *r* value | 0.33 | 0.29 | 0.46* | 0.38 |  |

***Note***. *, *p* < 0.05; **, *p* < 0.005. Bonferroni correction was used to correct the number of ROIs, *p* = 0.05/4 = 0.013; Abbreviations: Bi, bilateral; CMBs, cerebral microbleeds; MTG, middle temporal gyrus; mRS, modified Rankin Scale; LOC, lateral occipital cortex; PCU, precuneus.
